# The impacts of freeze–thaw cycles on saturated hydraulic conductivity and microstructure of saline–alkali soils

**DOI:** 10.1038/s41598-021-98208-0

**Published:** 2021-09-20

**Authors:** Wenshuo Xu, Kesheng Li, Longxiao Chen, Weihang Kong, Chuanxiao Liu

**Affiliations:** College of Water Conservancy and Civil Engineering, Shandong Agricultural University, Tai’an, 271018 Shandong China

**Keywords:** Civil engineering, Engineering, Environmental impact, Hydrology

## Abstract

Study on the microscopic structure of saline–alkali soil can reveal the change of its permeability more deeply. In this paper, the relationship between permeability and microstructure of saline–alkali soil with different dry densities and water content in the floodplain of southwestern Shandong Province was studied through freeze–thaw cycles. A comprehensive analysis of soil samples was conducted using particle-size distribution, X-ray diffraction, freeze–thaw cycles test, saturated hydraulic conductivity test and mercury intrusion porosimetry. The poor microstructure of soil is the main factor that leads to the category of micro-permeable soil. The porosity of the local soil was only 6.19–11.51%, and ultra-micropores (< 0.05 μm) and micropores (0.05–2 μm) dominated the pore size distribution. Soil saturated water conductivity was closely related to its microscopic pore size distribution. As the F–T cycles progressed, soil permeability became stronger, with the reason the pore size distribution curve began to shift to the small pores (2–10 μm) and mesopores (10–20 μm), and this effect was the most severe when the freeze–thaw cycle was 15 times. High water content could promote the effects of freeze–thaw cycles on soil permeability and pore size distribution, while the increase of dry density could inhibit these effects. The results of this study provide a theoretical basis for the remediation of saline–alkali soil in the flooded area of Southwest Shandong.

## Introduction

The problem of soil salinization is distributed in more than 100 countries and regions around the world. At present, the salinized soil has reached 1 billion hectares in the world and is gradually increasing every year^1,2^. Soil salinization will impoverish the land, which is not conducive to the health maintenance of the local ecosystem^3^. Salinization leads to low agricultural production capacity^4,5^, which has become one of the major problems restricting the development of global agricultural production and environmental construction. Studies have shown that soil salinization is essentially soil degradation, which will increase soil bulk density and electrical conductivity, and reduce soil permeability and water retention capacity^6,7^. On this basis, in order to improve this bad property, the current improvement methods are generally accepted to add desulfurization gypsum, biomass charcoal and fly ash to the saline–alkali soil^8–11^. However, the problems of cost control and environmental pollution cannot be ignored^12,13^. In order to explore a new idea of saline–alkali soil improvement, our study is devoted to studying the relationship between soil microstructure and its physical properties (this paper mainly refers to soil permeability) after freeze–thaw (F–T) cycles.

At present, there are about 340,000 km^2^ of saline–alkali land in China, accounting for 25.2% of the country's arable land area. These saline–alkali lands are mainly distributed in northern China, especially in the Yellow River Basin^14^. The Yellow River floodplain in the southwest of Shandong Province in China is a seasonal permafrost region. The saline–alkali land in this region is scattered and widely distributed, covering an area of more than 163 km^2^, accounting for 45.96% of the unused land^15^. The region relies mainly on water conservancy measures to control the saline–alkali land, but because of its inland location, fresh water resources are scarce. Although people in the area have been using straw returning to improve the saline land, the effect is still poor. Thus, it is urgent to develop a new saline–alkali land improvement measure.

Many scholars have proved that there is a close relationship between the physical properties and microstructure of soils^16–19^ analyzed the connection mode of particles in the skeleton of salinized soil and found that the cement of salt crystals in the skeleton would affect the mechanical strength of salinized soil. Zhang et al.^20^ by means of SEM (scanning electron microscope), MIP (mercury intrusion porosimetry) and NA (nitrogen adsorption test) to conduct a complete qualitative and quantitative evaluation of the pore characteristics of clay in coastal areas, and found that the colloid bonding and disordered open flocculating structure of the clay in Zhanjiang area contributed to the poor physical and mechanical properties of the clay in this area. Jha and Sivapullaiah^21^ studied the microstructure of clay by SEM and analyzed the physical properties of lime treated montmorillonite. MIP, which is a relatively straightforward method that can be used to quantitatively obtain reproducible values of pore size distribution. Nowadays, MIP analysis has gradually been introduced into the study of the microstructure of saline soil, and has become a common microstructure analysis method. Liu et al.^22^ successfully analyzed and explained the changes of microstructure of lime-stabilized saline soils by MIP analysis. Liu et al.^23^ used MIP to analyze the pore distribution of farmland saline–alkali soil in the Yellow River Delta, providing guidance for remediation, utilization and development of coastal saline–alkali soil in the Yellow River Delta. In addition, as one of the physical properties of soil, soil water permeability is used to characterize soil permeability and internal pore characteristics, and it is an important parameter in agricultural irrigation^24,25^. It has been proved that the microstructure of saline–alkali soil is closely related to its physical properties, especially its permeability^26^. Therefore, in the field of civil engineering, soil microstructure has gradually become one of the criteria for determining soil physical properties. However, research mainly focuses on loess and clay, and researches in the field of saline–alkali soil are relatively scarce. Therefore, the researches on the microstructure of saline–alkali soil can provide a new idea for the restoration of such degraded soil.

Recently, many scholars have found that F–T cycles can lead to changes in soil physical properties and microstructure. For unsaturated dispersed soils with different salinity, the F–T cycles could change their instantaneous water conductivity and matric suction, and the effects were not monotonic with the increase of the number of F–T cycles^27^. F–T cycles increased soil porosity and saturated water conductivity, change soil physical properties, and there is at least one cycle time threshold (between 5 and 20 cycles)^28^. Liu et al.^22^ found that the freeze–thaw cycles would destroy the coarse particles in lime solidified saline–alkali soil, collect fine particles and increase the diameter of macropores. Moreover, many researchers have studied the relationship between F–T cycles and soil permeability, and it is believed that dry density and water content are important factors affecting freeze–thaw cycles^20–31^. Meanwhile, the previous studies on soil after F–T cycles mostly focused on its mechanical properties and mostly qualitative research^32,33^. However, there are no comprehensive studies on the permeability and microstructure of saline–alkali soil in the southwest of Shandong Province after freeze–thaw cycles.

Inspired by the above studies, particle-size distribution (PSD) analysis, X-ray diffraction (XRD) analysis, freeze–thaw (F–T) cycle test, saturated hydraulic conductivity (SHC) test and mercury intrusion porosimetry (MIP) analysis were performed on the saline–alkali soil remolded according to different dry densities and water content in the flooded area of Southwest Shandong Province. The objective of this study is to determine the saturated hydraulic conductivity and microscopic pore size distribution of saline–alkali soil in the floodplain of Southwest Shandong Province after F–T cycles. It is expected to find the relationship between the microstructure and permeability of the saline–alkali soil in this area, so as provide a new theoretical basis for its treatment.

## Materials and methods

### Experimental materials

The test soil samples were collected in He-ze City, Shandong Province, which is located in the Yellow River Basin at an altitude of 50 m in the southwest of Shandong Province, and is a typical Yellow River flooding area in Shandong Province. Its region has a warm temperate monsoon continental climate with strong seasonality in temperature, rainfall, wind and evaporation. The average annual temperature in this region is 13.7 °C, the extreme minimum temperature is − 16.5 °C, and the extreme maximum temperature is 40.5 °C. It belongs to the seasonal frozen soil region. The annual frost-free period is 210 days, and the annual average precipitation is 625 mm, which is concentrated in June to September. The annual average wind speed is 1.95 m s^−1^, and the average wind speed from January to April is 2.33 m s^−1^. The annual average evaporation is 874.82 mm, the maximum value appears in June, and the minimum value appears in January. The area is inland and lacks fresh water, so the Yellow River is the only source of irrigation water.

The local soil was severely damaged by salt, and its EC_1:5_ reached 1.44 dS m^−1^, PH < 8.5, belonging to weakly alkaline soil. At the same time, exchange sodium percentage (ESP) reaches 31.14%, far more than 15%, so these poor soils are usually classified as “saline–alkali soil” (Table 1). The accumulation of salt, especially excessive Na^+^, led to the deterioration of soil properties in this area, with a salt content of 61.96 g kg^−1^. The *K*_*s*_ of the soil in this area was only 1.48 × 10^–5^ cm s^−1^, which belongs in the micro-permeable water class according to the code for engineering geological investigation of water resources and hydropower^33^.

**Table 1:** Basic physicochemical properties of the saline–alkali soils used in this study.

Silty clay			
Soil property		Soil property	
Bulk density (g cm^−3^)	1.54	Dry density (g cm^−3^)	1.35
Optimum water content (%)	16.00	Maximum dry density (g cm^−3^)	1.58
Electrical conductivity (dS m^−1^)	1.44	Ca^2+^ (mg/100 g)	10.64
Soluble salt (g kg^−1^)	6.20	Mg^2+^ (mg/100 g)	3.09
PH	7.62	K^+^ (mg/100 g)	1.87
Plastic limit (%)	21.27	Na^+^ (mg/100 g)	17.75
Liquid limit (%)	34.62	Clay (%)	4.95
Saturated hydraulic conductivity (10^−5^ cm s^−1^)	1.48	Silt (%)	42.90
		Sand (%)	52.15
Exchangeable sodium percentage (ESP)	31.14	Sodium adsorption ratio (SAR)	21.43

**Figure 1:**
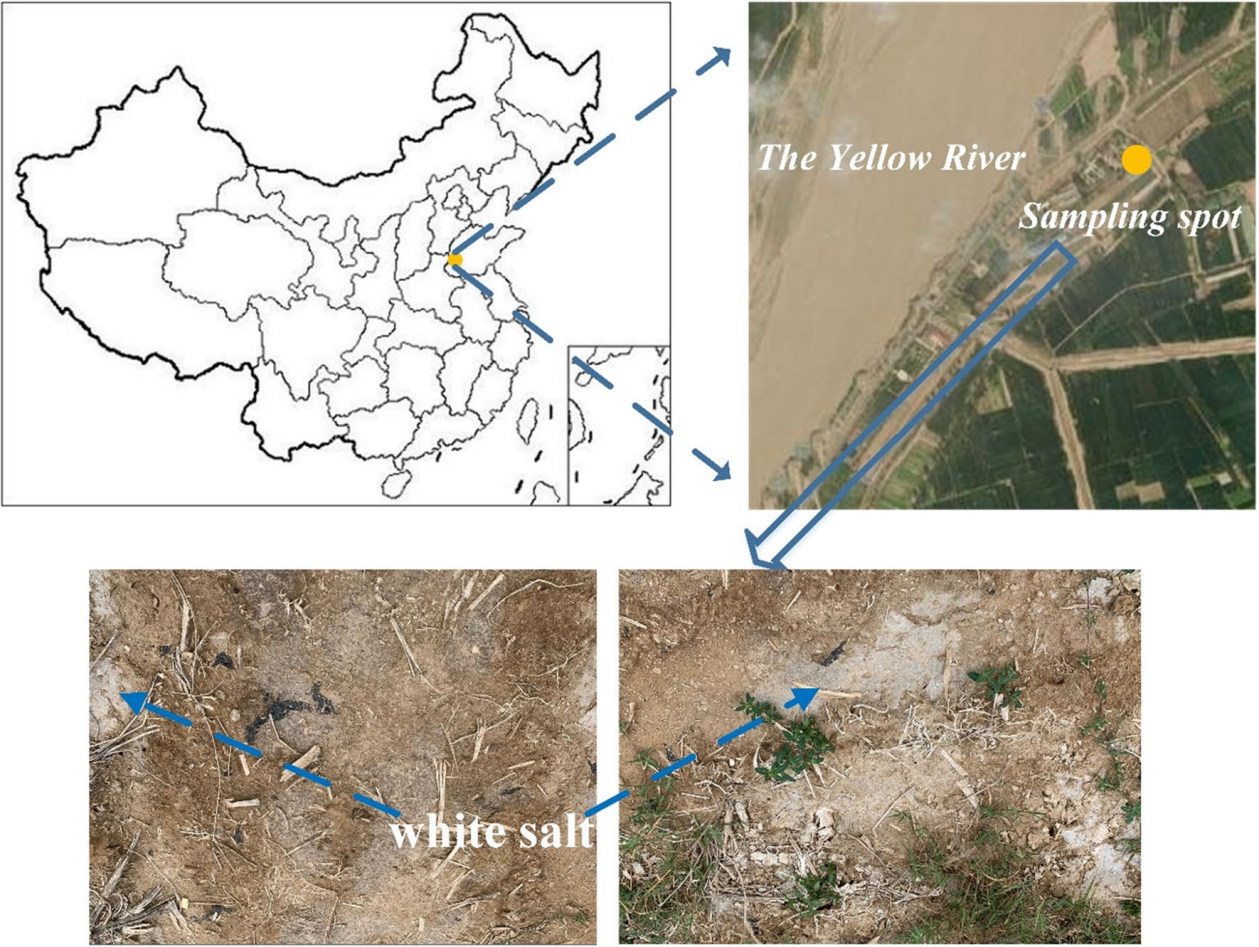
Study area and sampling location.

Soil samples were collected from the vicinity of Dongming County, He-ze City, Shandong Province, located at 115.08° E, 35.38° N (Fig. 1). A number of undisturbed soil samples and scattered soil samples were taken from the test site for later measurement of the basic physical properties of the soil and preparation of soil samples for subsequent experiments. The clay (< 0.002 mm) in the soil samples is 4.95%, and the silt (0.002–0.02 mm) is 42.90%, sand (> 0.02 mm) in soil, reaching 52.15%. The soil texture of this region can therefore be classified as silty clay. Other basic physicochemical properties of the soil in this area are shown in Table 1.

### Preparation of specimens

A small number of soil samples were dried at 105 °C, crushed and sifted through 75 μm for PSD and XRD analysis. The other samples were air-dried, rolled and crushed and passed through a 2 mm sieve. The F–T cycles (*N*) of 0, 1, 5, 10, 15, 20 were set. The water content was 13%, 16%, 19%, and the dry density of remolded soil was 1.48 g cm^−3^, 1.53 g cm^−3^, 1.58 g cm^−3^, a total of 54 groups of samples (Table 2). The remolded sample were left in the shade for 24 h to ensure sufficient moisture diffusion. Then, the 77 × 40 mm soil sample was prepared by electrohydraulic demodding mechanism. The 61.8 × 40 mm ring knifes coated with vaseline were used to cut the samples and wraped with the plastic film for F–T cycle test and saturated hydraulic conductivity test. At the end of the above tests, we gently removed the soil samples from the ring knifes and prepare the test samples for MIP analysis according to the liquid nitrogen vacuum freeze-drying method^34,35^. The soil samples were cut into 5 × 5 × 10 mm cubes for MIP analysis.

**Table 2:** The sample group in this study.

Freeze–thaw cycles	Water content (%)	Dry density (g cm^−3^)		
		1.48	1.53	1.58
0	13	A1	A2	A3
	16	A4	A5	A6
	19	A7	A8	A9
1	13	B1	B2	B3
	16	B4	B5	B6
	19	B7	B8	B9
5	13	C1	C2	C3
	16	C4	C5	C6
	19	C7	C8	C9
10	13	D1	D2	D3
	16	D4	D5	D6
	19	D7	D8	D9
15	13	E1	E2	E3
	16	E4	E5	E6
	19	E7	E8	E9
20	13	F1	F2	F3
	16	F4	F5	F6
	19	F7	F8	F9

A1 is freeze–thaw cycle 0 times, water content 13%, dry density 1.48 g cm^−3^.

### Particle size distribution and X-ray diffraction before F–T cycles

The PSD analysis of soil can facilitate us to understand the composition of soil particles and the uniformity of particle size distribution. The BT-9300S laser particle analyzer (Dandong Baite Instrument Co., Ltd., Dandong City, China) was used to complete soil PSD analysis, and two grading indexes were used to analyze the uniformity of soil particles, namely uniformity coefficient (*C*_*u*_) and curvature coefficient (*C*_*c*_). Uniformity coefficient is used to reflect the distribution of different particle groups, and *C*_*c*_ is used to judge the continuity of slope of cumulative curve of soil particle size distribution, and it is also an important index to judge whether soil particle size distribution is good. The expressions are as follows:

1 $${C}_{u}=\frac{{d}_{60}}{{d}_{10}}$$

2 $${C}_{c}=\frac{{d}_{30}^{2}}{{d}_{10}\times {d}_{60}}$$

where *d*_*10*_ is effective grain size, *d*_*30*_ is median grain size, and *d*_*60*_ is control grain size. *d*_10_, *d*_30_, *d*_60_ represent the percentage of particles of the corresponding size that are 10%, 30%, and 60% smaller than the sample.

**Figure 2:**
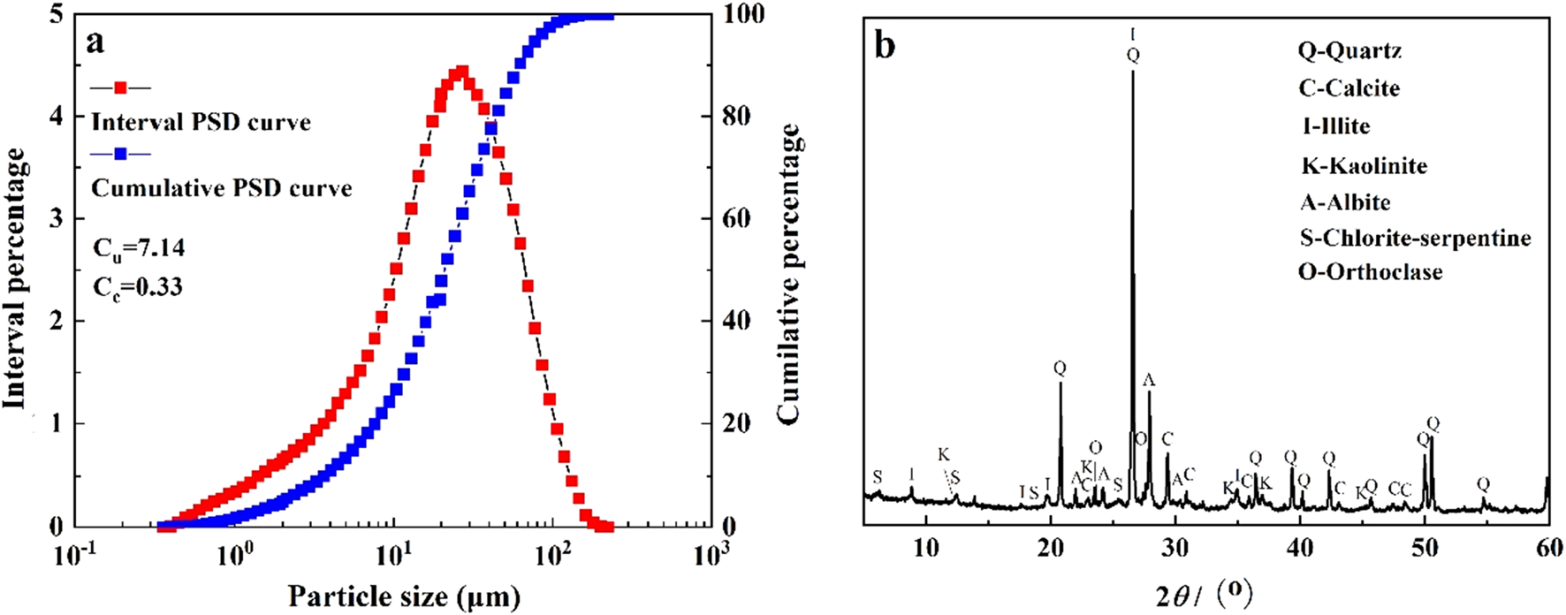
PSD curves and XRD curves of saline–alkali soils in the floodplain of southwestern Shandong Province: (**a**) particle size distribution cures and (**b**) X-ray diffraction pattern.

It is found that the particle size of saline–alkali soil in this area is in the range of 1–100 μm, and the particle density curve was unimodal (Fig. 2). The relevant soil characteristic parameters in this area, *C*_*u*_ = 7.14, *C*_*c*_ = 0.33. According to the regulations of the Ministry of Water Resources of the People's Republic of China^36^, *C*_*u*_ > 5, the distribution of soil grain size is not uniform. *C*_*c*_ < 1, indicating that the soil is dominated by small particles. From this, we can preliminarily judge that the grain size distribution of saline–alkali soil in this area is poor, leading to its poor permeability.

The mineral composition of soil affects the physical and chemical properties of soil^37,38^, which we analyzed using a TD-3500 X-ray diffractometer (Dandong Tongda Technology Co., Ltd., Dandong City, China). The semi-quantitative analysis of soil minerals was carried out using Jade 6.5 software.

Figure 2 shows the XRD map of the saline–alkali soil in this area. Table 3 shows the mineral composition of the local saline–alkali soil. The mineral composition of the soil includes quartz, albite, orthoclase, calcite, kaolinite, illite and chlorite. Primary minerals account for more than 90% of the total minerals, and quartz is the highest, accounting for 51.85% of the total minerals, followed by albite and calcite. Clay minerals (kaolinite, illite, chlorite) only accounted for 9.55% of the total mineral content of the soil, among which illite was dominant, reaching 5.68% of the total mineral content of the soil.

**Table 3:** The mineral composition of the saline–alkali soils in this study (%).

Mineral	Proportion (%)	
*Primary mineral*		
Quartz	51.85	90.45
Albite	22.61	
Orthoclase	2.87	
Calcite	13.12	
*Clay mineral*		
Kaolinite	0.83	9.55
Illite	5.68	
Chlorite	3.04	

### Freeze–thaw cycling experiments

Freezing and thawing will change the physical properties and internal structure^39^ of soil. F–T tests were carried out in a closed system and no moisture was supplied to the sample during the test for providing conditions similar to field conditions^40^. In this study, the freeze–thaw cycles were split into six: the initial state (no cycle) and sets ending after the 1st, 5rd, 10th, 15th and 20th cycle. The soil samples were subjected to a freezing temperature of − 15 °C for 12 h in order to obtain complete frost penetration. After freezing, they were allowed to thaw at a room temperature of about 10 °C for 12 h before being subjected to the next cycle. An ultra-low temperature refrigerated storage tank (DW-HL290) manufactured by Zhongke Meiling Cryogenics Co., Ltd was used for this purpose. On the basis of no more than 0.5 °C error, the refrigerated storage tank has a temperature adjustment range of − 5 to − 87 °C and the temperature is visible.

### Saturated hydraulic conductivity

Saturated hydraulic conductivity (*K*_*s*_) represents the upper limit of water migration rate for soil, and it is one of the crucial hydraulic properties of soil^41^. Soil permeability is closely related to its microstructure^42^. In this study, according to the code for geotechnical testing^43^, variable head osmotic apparatus (TST-55) was used for the SHC test. Before the test, the soil samples were placed in a vacuum saturator for 24 h to ensure that the soil samples reached the saturation state. The flow rate was measured under the condition of variable head. Considering the effect of temperature on water viscosity, the water temperature was monitored in real time during the test. The expression of *K*_*s*_ is as follows^44^:

3 $${K}_{s}=\frac{aL}{At}\mathit{ln}\frac{{h}_{1}}{{h}_{2}}$$

where *K*_*s*_ is the saturated hydraulic conductivity (cm s^−1^), *a* is the cross-sectional area of the standpipe (m^2^), *L* is the length of the sample (m), *A* is the cross-sectional area of the sample (m^2^), *h*_1_ and *h*_2_ are the initial and final water head with respect to the outflow (m) and *t* is the time for the hydraulic head difference to decrease from *h*_1_ to *h*_2_.

After using formula (3) to figure out *K*_*s*_, use ${k}_{20}=\frac{{\eta }_{T}}{{\eta }_{20}}$ to calculate *K*_*S*_ at standard temperature, where η_T_ is the dynamic viscosity coefficient of water at T °C and η_20_ is the dynamic viscosity coefficient of water at 20 °C.

### Mercury intrusion porosimetry

MIP is a relatively straightforward method, which can be used to derive the values of other important characteristic parameters, including total porosity, average pore diameter, median pore diameter, and most probable pore diameter, and the measured pore diameters range from 7 to 350 μm.

In present study, we measured soil porosity and pore size distribution using MIP. Pre-lyophilized MIP samples were directly used for the MIP analysis, we used a fully automatic PM-33-18 mercury intrusion analyzer (Conta Instruments Company, USA). Mercury will be injected into the soil sample under a certain degree of pressure. Therefore, according to the pressure applied to mercury and the volume of mercury invasion, the pore distribution of the soil sample can be obtained through the Washburn equation^45^. The expression is as follows:

4 $$p=-\frac{2\sigma \mathit{cos}\theta }{r}$$

where *P* is the applied pressure, *σ* is the surface tension coefficient of the immersed liquid, *θ* is the contact angle between liquid and solid materials, and *r* is the cylindrical pore radius. Among them, the values of *σ* and *θ* were 0.485 N m^−1^ and 140° in this study, respectively^46^.

Soil pores can be classified into inactive pores, capillary pores and aerated pores according to their function and size. The porosity of the inactive pore is less than 2 μm, and the permeability is the worst. As the diameter increases, the permeability of pores becomes better^20^. In order to facilitate the pore size classification of saline–alkali soil in the Yellow River floodplain of Southwest Shandong, China. Our study based on the Shear pore size division theory^47^, and other scholars^48,49^, combining with the pore characteristics of the local saline–alkali soil (Table 4) to divide the pore size of saline–alkali soil in this area.

**Table 4:** Classification of pore types in the saline–alkali soils in this study.

Pore type		Pore diameter (*d*) range (μm)	Pore composition
Aeration pore	Macropore	d ≥ 20	Inter-granular pores and parts of intra-granular pores
Capillary pore	Mesopore	10 ≤ d < 20	Intra-granular pores
	Small pore	2 ≤ d < 10	Inter-granular pores and parts of intra-granular pores
Inactive pore	Micropore	0.05 ≤ d < 2	Intra-granular pores
	Ultra-micropore	d < 0.05	Intra-granular pores

## Results

### Surface fragmentation of soil samples after F–T cycles

**Figure 3:**
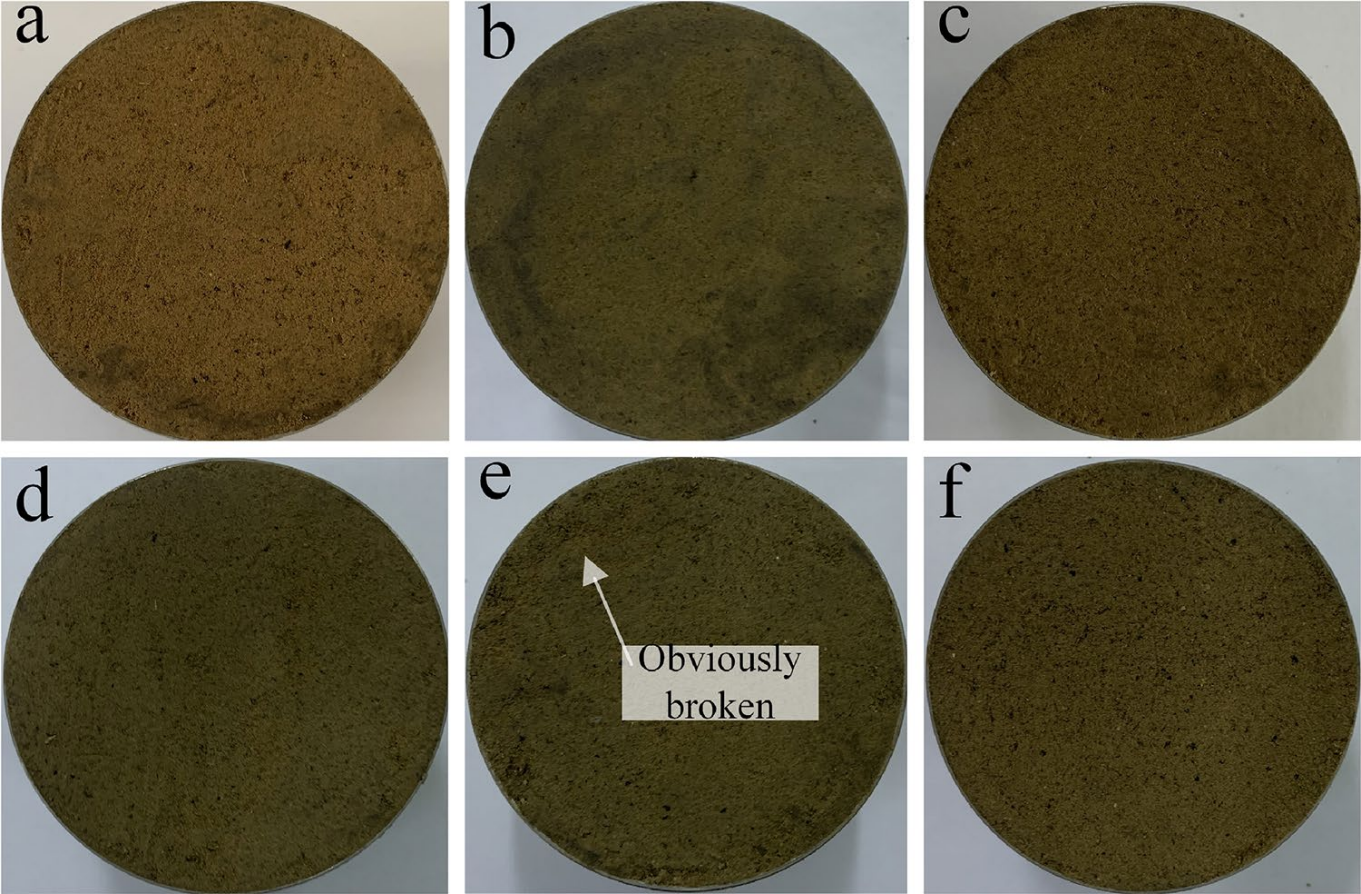
Soil samples with a moisture content of 13%, 1.48 g cm^−3^ and different freeze–thaw cycles: (**a**) *N* = 0, 13%, 1.48 g cm^−3^ (A1), (**b**) *N* = 1, 13%, 1.48 g cm^−3^ (B1), (**c**) *N* = 5, 13%, 1.48 g cm^−3^ (C1), (**d**) *N* = 10, 13%, 1.48 g cm^−3^ (D1), (**e**) *N* = 15, 13%, 1.48 g cm^−3^ (E1) and (**f**) *N* = 20, 13%, 1.48 g cm^−3^ (F1).

**Figure 4:**
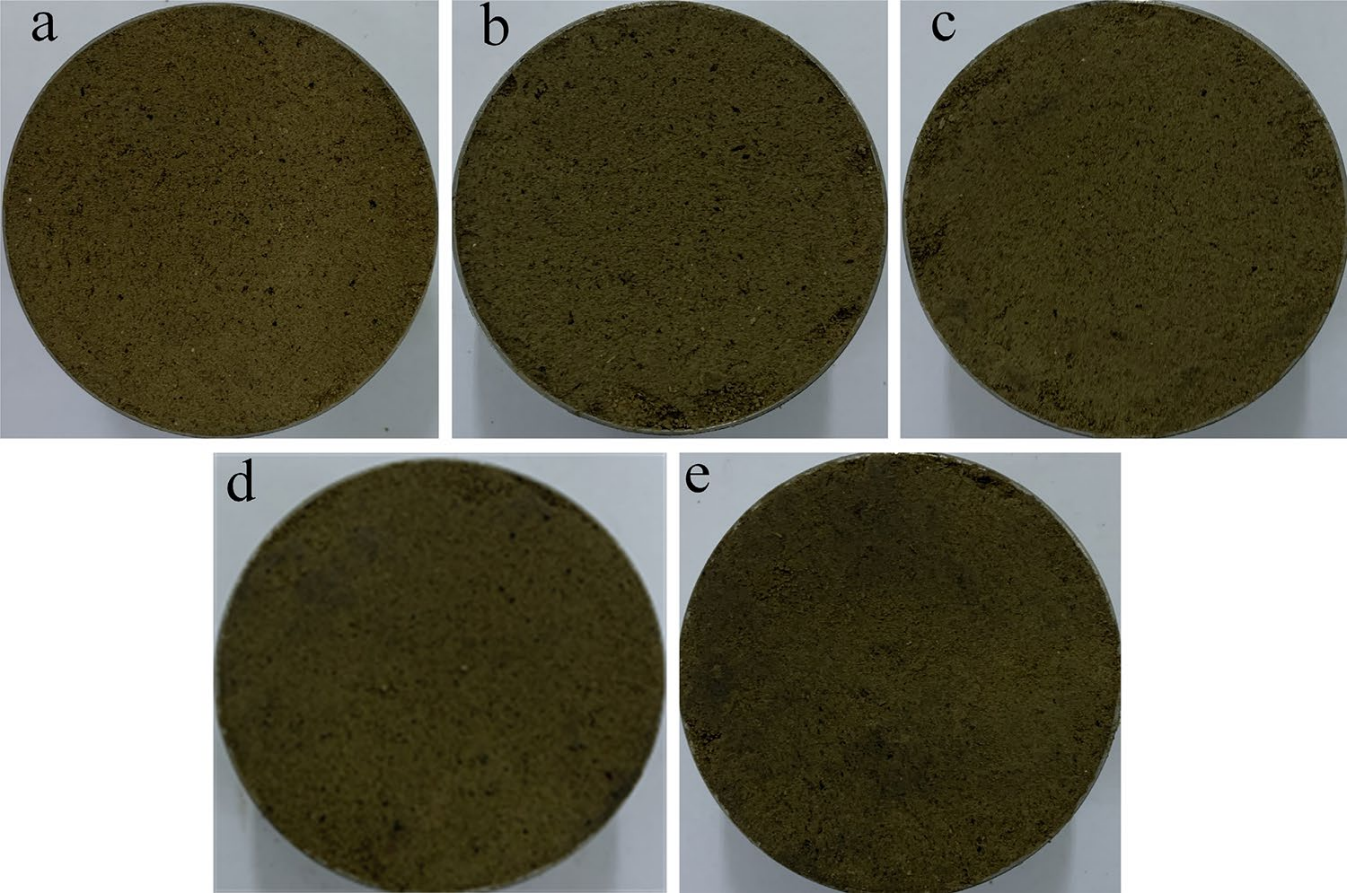
Soil samples affected by a single factor after 20 freeze–thaw cycles. (**a**) *N* = 20, 13%, 1.48 g cm^−3^ (F1), (**b**) *N* = 20, 16%, 1.48 g cm^−3^ (F4), (**c**) *N* = 20, 19%, 1.48 g cm^−3^ (F7), (**d**) *N* = 20, 13%, 1.53 g cm^−3^ (F2) and (**e**) *N* = 20, 13%, 1.58 g cm^−3^ (F3).

**Figure 5:**
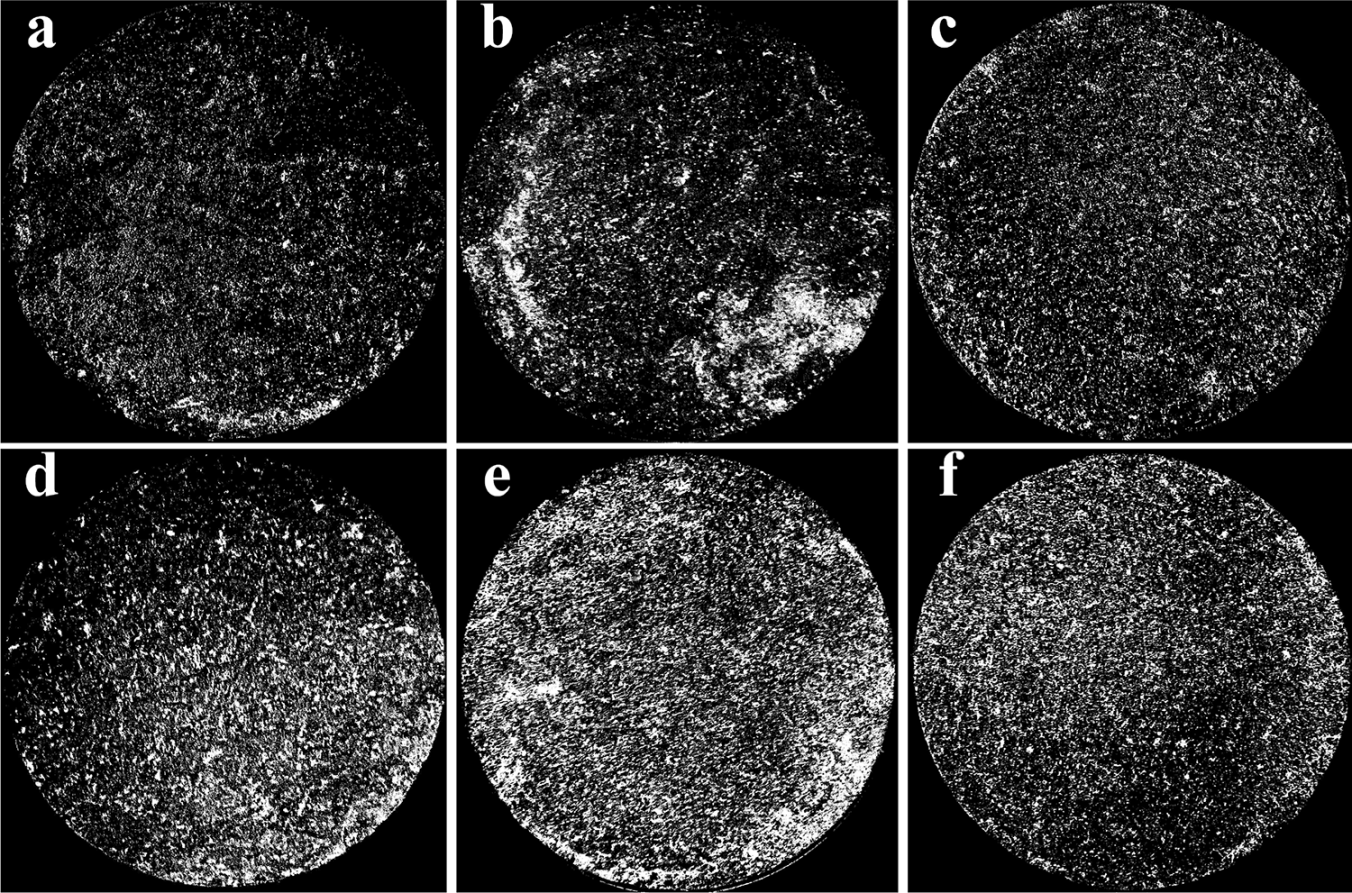
Binary images of soil samples with a moisture content of 13%, 1.48 g cm^−3^ and different freeze–thaw cycles: (**a**) *N* = 0, 13%, 1.48 g cm^−3^ (A1), (**b**) *N* = 1, 13%, 1.48 g cm^−3^ (B1), (**c**) *N* = 5, 13%, 1.48 g cm^−3^ (C1), (**d**) *N* = 10, 13%, 1.48 g cm^−3^ (D1), (**e**) *N* = 15, 13%, 1.48 g cm^−3^ (E1) and (**f**) *N* = 20, 13%, 1.48 g cm^−3^ (F1).

**Figure 6:**
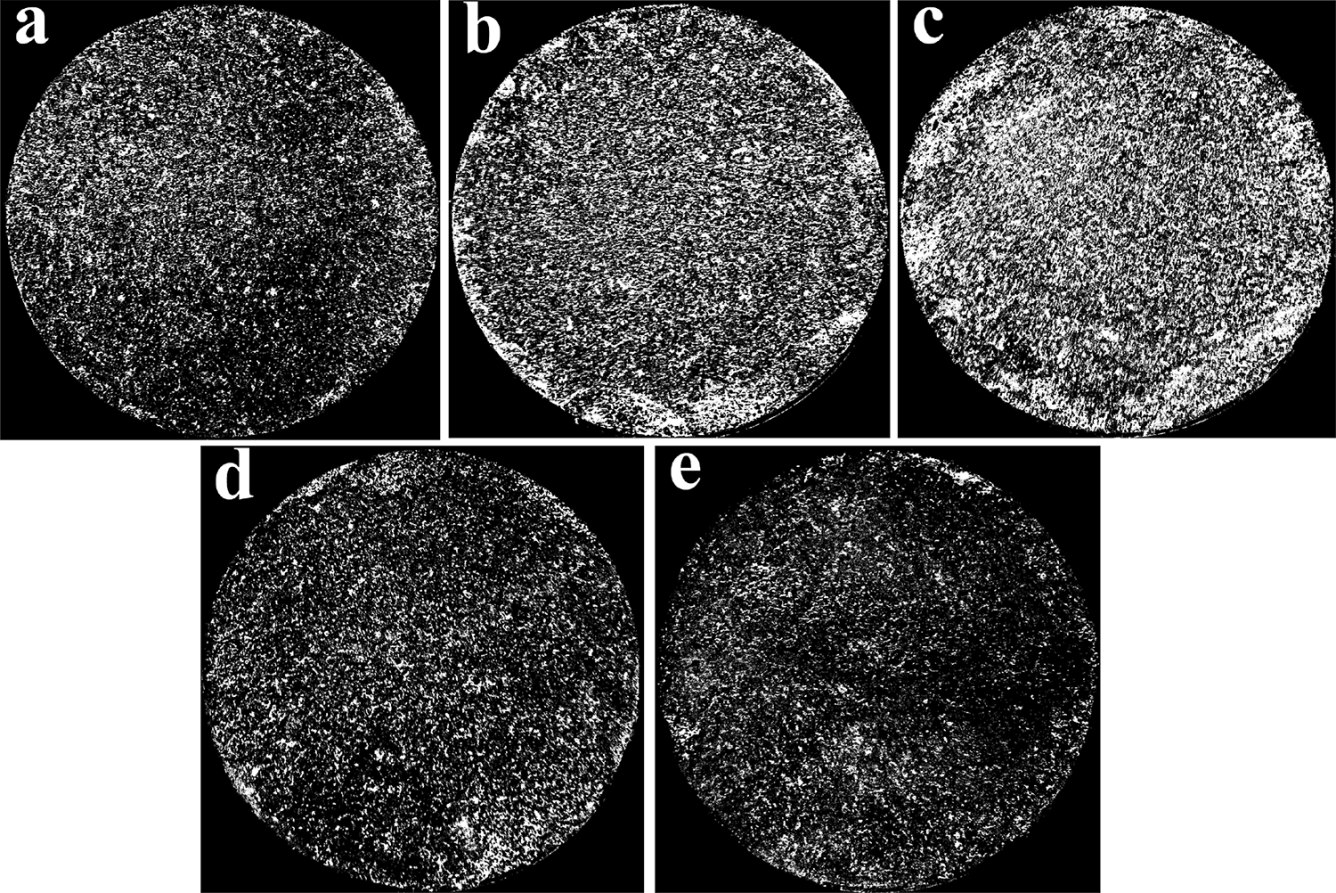
Binary images of soil samples affected by a single factor after 20 freeze–thaw cycles. (**a**) *N* = 20, 13%, 1.48 g cm^−3^, (F1) (**b**) *N* = 20, 16%, 1.48 g cm^−3^ (F4), (**c**) *N* = 20, 19%, 1.48 g cm^−3^ (F7), (**d**) *N* = 20, 13%, 1.53 g cm^−3^ (F2) and (**e**) *N* = 20, 13%, 1.58 g cm^−3^ (F3).

In this study, in order to better observe the macroscopic effects of freeze–thaw cycles on soil, we selected several representative experimental groups to demonstrate. Figure 3 shows soil samples with a water content of 13%, 1.48 g cm^−3^ and different freeze–thaw cycles. With the increase of the number of freeze–thaw cycles, we found that the surface fragmentation of soil samples from A1 to F1 was more and more obvious. By observing F1–F3 (Fig. 4), it is found that under the same number of freeze–thaw cycles and dry density, high water content can make the surface of soil samples more broken. However, the change of dry density of a single factor has no obvious influence on the surface macrophenomena of soil samples (Fig. 4). In order to highlight their differences more intuitively, we made Figs. 3 and 4 binary image respectively to obtain Figs. 5 and 6. In the selected circular area in the figure, black is the soil matrix and white is the broken surface. Based on Figs. 5 and 6, we found that the area variation of white area was basically consistent with the variation law of the crushing surface in Figs. 3 and 4. At the same time, the Image J software was used to calculate the fractal dimension (Table 5). The fractal dimension of pores mainly reflects the irregularity and complexity of the contact boundary between pores and solid particles. The larger the value, the more complex the distribution of pores and the more irregular the boundary between pores and solid particles. With the increase of the number of freeze–thaw cycles, the fractal dimension reaches the maximum at *N* = 15. It also increases with the increase of water content and decreases with the increase of dry density.

**Table 5:** The fractal dimension of soil samples.

Sample Number	A1	B1	C1	D1	E1	F1	F2	F3	F4	F7
*Soil samples*										
D	1.459	1.601	1.622	1.709	1.801	1.676	1.627	1.567	1.818	1.868

D is the fractal dimension.

A1 is freeze–thaw cycle 0 times, water content 13%, dry density 1.48 g cm^−3^.

### Saturated hydraulic conductivity after F–T cycles

**Figure 7:**
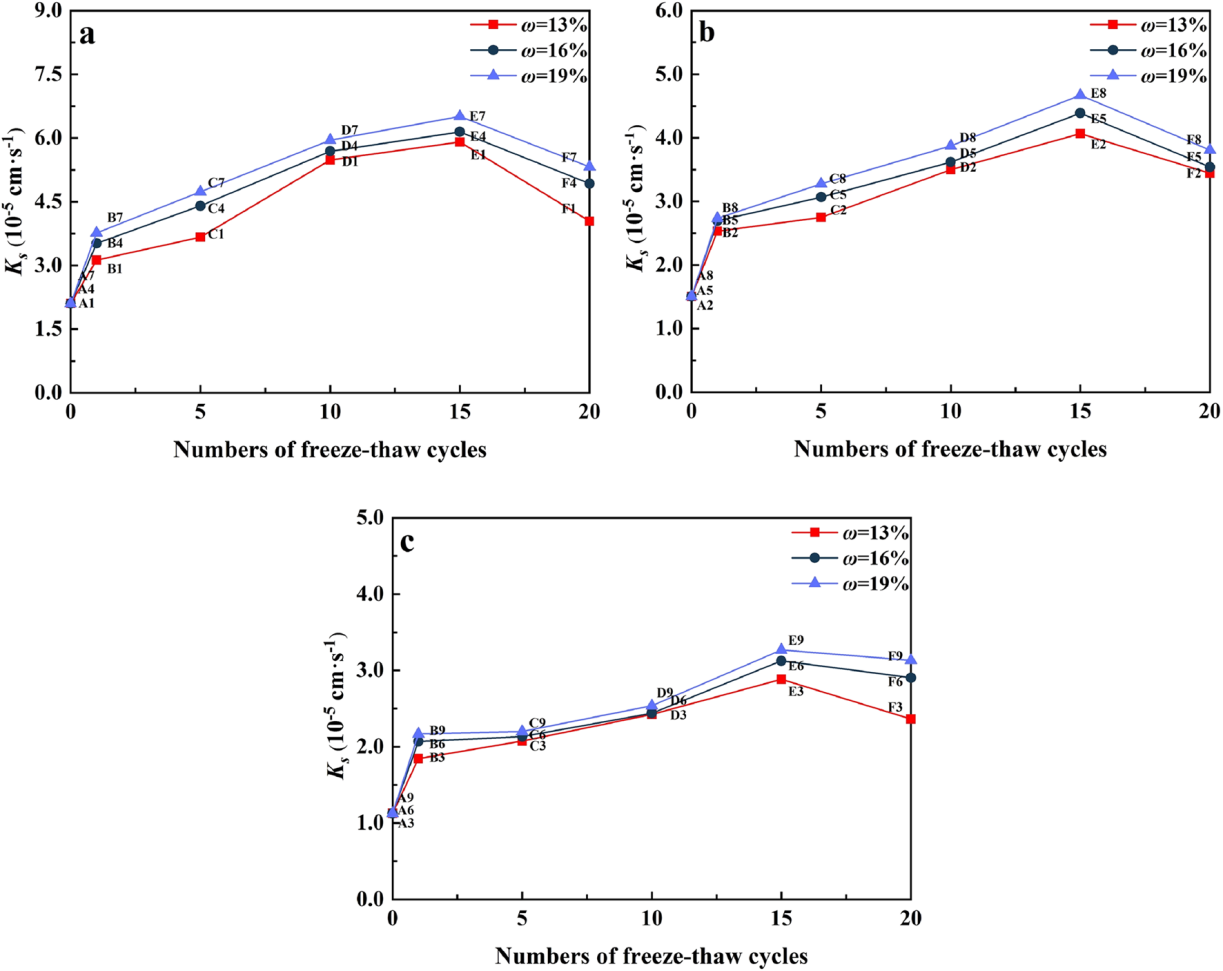
The changes of *K*_*s*_ of water-bearing remolded soils at the study site in the floodplain of southwestern Shandong Province caused by the changes of F–T cycles: (**a**) *ρ*_*d*_ = 1.48 g cm^−3^, (**b**) *ρ*_*d*_ = 1.53 g cm^−3^ and (**c**) *ρ*_*d*_ = 1.58 g cm^−3^.

Figure 7 summarizes the changes of *K*_*s*_ of water-bearing remolded soil caused by the changes of F–T cycles. Under the three dry densities of 1.48 (Fig. 7a), 1.53 (Fig. 7b) and 1.58 g cm^−3^ (Fig. 7c), the soil *K*_*s*_ increased with the increase of the number of cycles and reached the threshold value at *N* = 15, and then the increasing pressure generated by the expanding ice crystals reduced the porosity inside the soil, *K*_*s*_ showed a decreasing trend.

**Figure 8:**
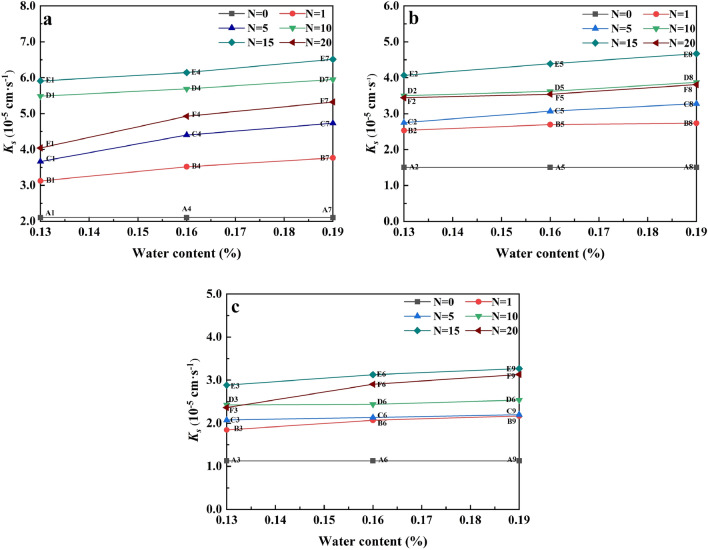
The changes of *K*_*s*_ with water content of water-remolded soils at the study site in the floodplain of southwestern Shandong Province: (**a**) *ρ*_*d*_ = 1.48 g cm^−3^, (**b**) *ρ*_*d*_ = 1.53 g cm^−3^ and (**c**) *ρ*_*d*_ = 1.58 g cm^−3^.

Taking 1.48 g cm^−3^ as an example (Fig. 7a), when *N* = 15, the *K*_*s*_ of the three water contents of soil samples increased by 260.95%, 270.95% and 283.33%, respectively. We found that after the first cycle, the increment of *K*_*s*_ decreased with the change of the cycle. Nevertheless, the increment of the first cycle was the largest, which might be because the freeze–thaw cycle affected the development of soil pores. Figure 8 shows the changes of *K*_*s*_ with water content of water-remolded soil. F–T cycle before (N = 0) and after freezing and thawing cycle (N > 0) of soil samples have significant differences. When *N* = 0, the *K*_*s*_ was hardly affected by the water content changes. After freezing and thawing cycle, *K*_*s*_ of soil had approximate linear relationship with the increase of water content, the F–T cycle test with us observed is consistent with the regular pattern of soil sample surface broken. However, the soil water content increased from 13 to 19%, and the increase of *K*_*s*_ was relatively small. Under the three dry densities, the maximum elevation of *K*_*s*_ was 31.68% (Fig. 8a), 18.91% (Fig. 8b) and 32.63% (Fig. 8c) respectively. At the same time, we found that the *K*_*s*_ of soil samples would be enhanced as long as they experienced F–T cycles, which indicated that F–T cycles would have an impact on soil structure.

**Figure 9:**
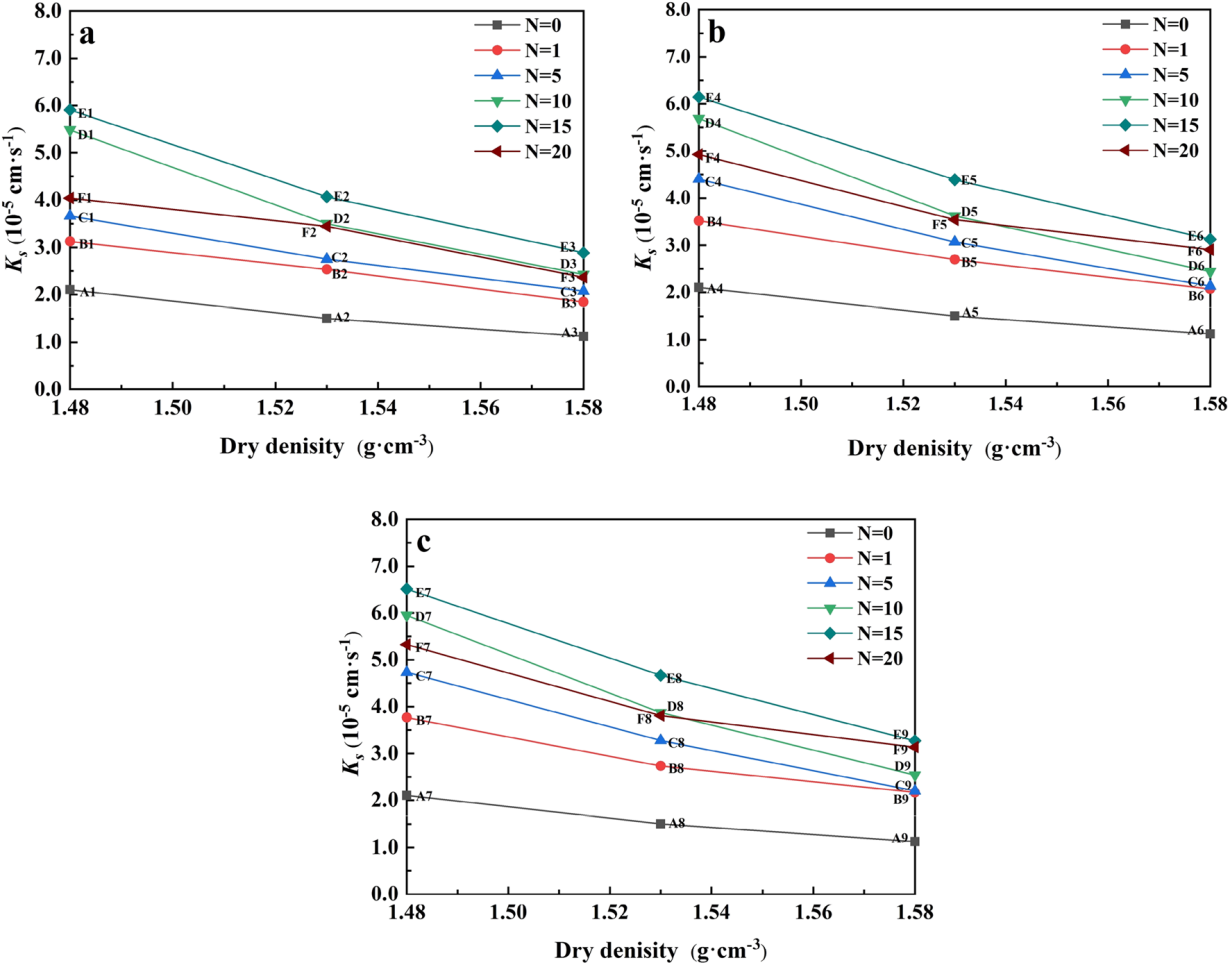
The changes of *K*_*s*_ with dry density of water-remolded saline–alkali soils at the study site in the floodplain of southwestern Shandong Province: (**a**) *ω* = 13%, (**b**) *ω* = 16% and (**c**) *ω* = 19%.

The relationship between *K*_*s*_ and dry density of water-bearing remolded saline–alkali soil is shown in Fig. 9. When the soil dry density is high, the *K*_*s*_ decreases significantly, but the attenuation rate decreases. The higher the dry density of the soil, the higher the degree of internal consolidation of the soil, which will affect the pores in the soil, resulting in compression or even closure, thus blocking the seepage channel. In addition, we found that compared with the F–T cycles of 20 times (*N* = 20) and 10 times (*N* = 10), with the increase of dry density, the saturated water content curves of both will present a crossover phenomenon, and this crossover phenomenon is more obvious with the increase of water content.

### Quantitative analysis of micropore characteristics

**Figure 10:**
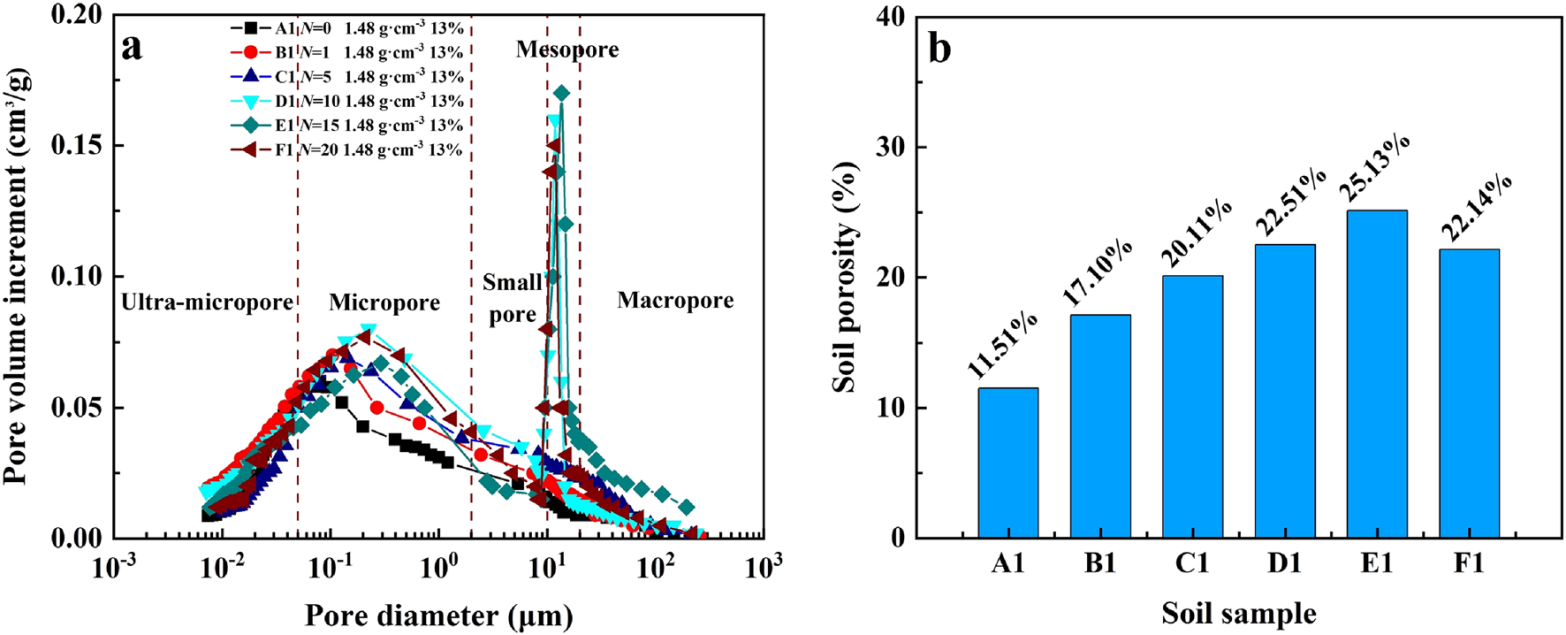
Pore-size distribution cures and bar chart of soil porosity distribution for soils at the study site in the floodplain of southwestern Shandong Province: (**a**) The water content is 13% and the dry density is 1.48 g cm^−3^, pore-size distribution curves with different F–T cycles. (**b**) Bar chart of soil porosity distribution.

Figure 10a summarizes the pore size distribution of soil samples subjected to F–T cycles under the same dry density and water content conditions, and the pore diameter corresponding to the peak point in the curve is the most likely pore diameter. The soil samples with 13% moisture content and 1.48 g cm^−3^ dry density had more ultra-micropores (< 0.05 μm) and micropores (0.05–2 μm) and less macropores (≥ 20 μm) after F–T cycles, but the results were higher than those of the soil samples without freezing–thawing cycle (*N* = 0). We found that the peak value of pore size distribution curve shifts to the right and increases with the freeze–thaw cycle. After 10 F–T cycles, the curve presents a bimodal distribution, and the pore size distribution of the second peak is more than 10 μm. When the freeze–thaw cycle reaches 15 times (E1), the peak value reaches the highest, and the porosity reaches 32.39% (Fig. 10b). Although the peak value of F1 is lower than that of E1, it is still significantly higher than that of A1. Among them, the first peak of E1 was 19.40% and 14.93% less than that of D1 and F1. The second peak was 6.25% and 13.33% higher than D1 and F1, respectively. This indicates that the second peak in the pore diameter distribution curve plays a decisive role after 10 F–T cycles. The distribution curves of D1 and F1 approximately coincide, and the porosity difference between them is less than 3%, which is consistent with the result of saturated water conductivity.

**Figure 11:**
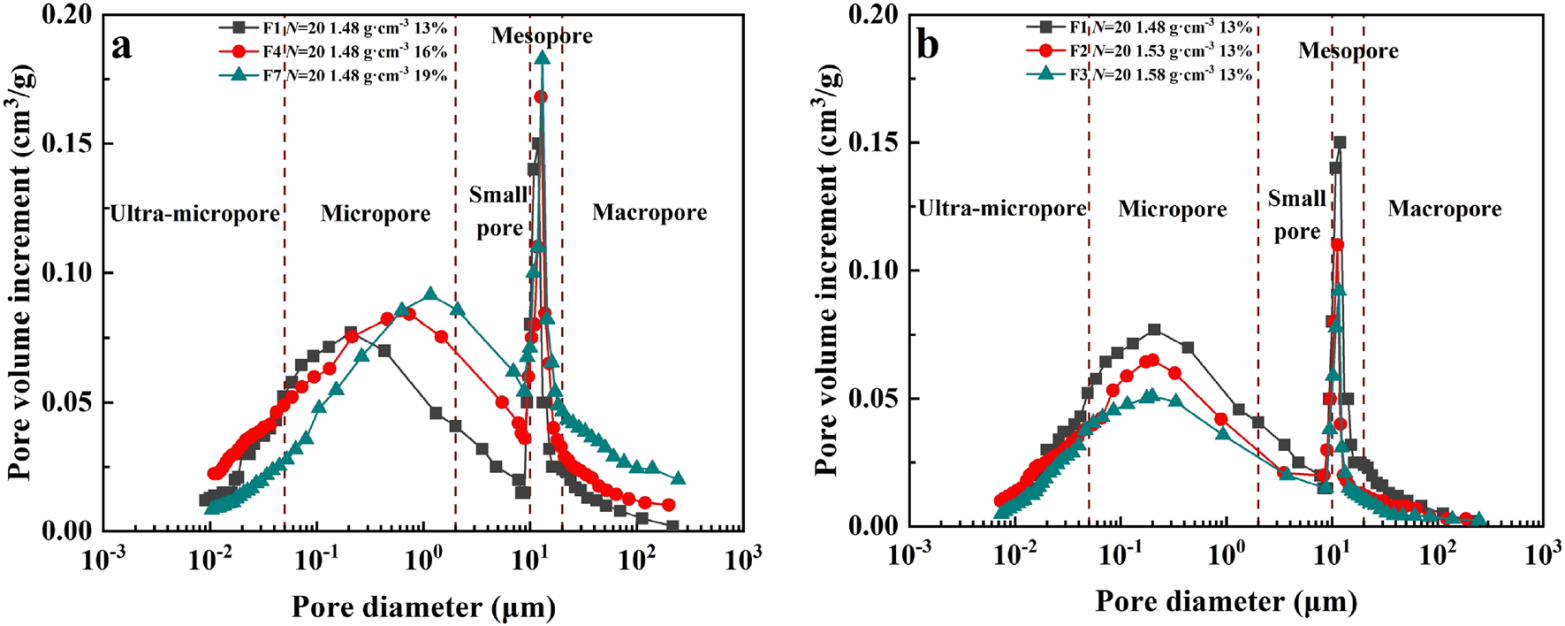
Pore-size distribution cures with different water content and dry density or soils at the study site in the floodplain of southwestern Shandong Province: (**a**) The dry density is 1.48 g cm^−3^, pore-size distribution cures with different water contents. (**b**) The water content is 19%, pore-size distribution cures with different dry densities.

Figure 11 shows the influence of water content and dry density on pore size distribution of soil samples. With the increase of soil water content, the dominant pore size moved to the right, gradually changing from micropores (0.05–2 μm) to pores (2–10 μm), and the content increased. On the contrary, the distribution of dominant pore size is not affected by the change of dry density, and the content will decrease with the increase of soil dry density. When the dry density increases from 1.48 to 1.58 g cm^−3^, the first peak and second peak of dominant pore size decrease by 36.75% and 13.25% respectively. These results indicate that the *K*_*s*_ of soil is closely related to its microstructure, while water content and dry density also affect the microstructure of soil samples after F–T cycles to varying degrees, and thus affect the *K*_*s*_ of soil.

## Discussion

We speculate that the degradation of permeability of saline–alkali soil is closely related to its microstructure. Therefore, the saturated water conductivity test of soil was carried out, and PSD, XRD, MIP and other advanced technologies were adopted to study the microstructure of saline–alkali soil. The relationship between microstructure and permeability of saline–alkali soil after freeze–thaw cycle was analyzed in order to provide a theoretical basis for the restoration of microstructure of saline–alkali soil.

### Particle composition and mineral composition of saline–alkali soil based on PSD and XRD

Soil particle composition is an important factor determining soil structure characteristics^50^. The distribution and texture of soil particle size directly affect the basic physical properties of soil, especially the water absorption capacity of soil^51^. The mineral composition of the soil is also one of the important factors affecting the soil permeability. The high proportion of clay and clay minerals will reduce the soil permeability due to their hydrophilicity^14,20,52^. Our research shows that clay (particle size < 0.002 mm) only accounts for 4.95% in saline–alkali soil, and the proportion of clay minerals is 9.55%, both of which account for less than 10% of the total proportion.

A study found that the permeability of saline soil in the Yellow River basin decreases with the increase of clay particle content^53^, and added Yellow River sediment to change the particle size distribution to remediate the saline–alkali soil. In addition, high clay mineral content can reduce soil permeability, and soil weathering degree is low when clay mineral content is less than 15%^23^. The soil samples used in this study had low content of clay particles and clay minerals. Based on these, we focused on the microstructure of soil and explored the microstructure of soil. Thus, MIP analysis is very necessary in our research.

### Saturated hydraulic conductivity of saline–alkali soil based on freeze–thaw cycling experiments

The freeze–thaw cycle is a process of energy input and output^54^, in which the transport of water and salt will lead to changes in soil grain size and structure^50^. The degree of soil weathering by freezing is related to the number of freeze–thaw cycles and water content. After the initial freeze–thaw cycles, the crushing of coarse particles and the aggregation of fine particles in the soil will lead to a significant increase in porosity. After more freeze–thaw cycles, the soil structure will gradually aggregate and the particle size distribution tends to be stable^50^. According to the local daily freezing and thawing times, we set a total of 6 freeze–thaw cycles (including *N* = 0). It was found that the soil permeability coefficient increased non-linearly with the increase of the number of cycles. The reason is that ice lenses gradually form during freezing, and ice melts in the frozen soil during thawing, creating cavities that act as channels and increase soil permeability^55^. When the number of F–T cycles reaches 15 times, it reaches the peak value. Compared with *N* = 0, the *K*_*s*_ increases by 209.77%.

The initial porosity of soil plays a decisive role before F–T cycles^29^, and the initial dry density of soil affects the initial porosity. Water to ice produces a volume change of about 9%, which affects the volume expansion of the pores during freezing^56^. Therefore, according to the standard compaction test, we determined the maximum dry density of the saline–alkali soil of 1.58 g cm^−3^ and the optimal water content of 16%, and the floating value can meet the conditions of the test area coverage. After the F–T cycles, soil is coupled by salt heave and frost heave, and soil pores change, resulting in more internal seepage channels, which means that soil porosity is the main influencing factor leading to the change of saturated water content. Freeze–thaw soils with high water content produce more expansive ice, which breaks the bonds between particles, in contrast, when soil water content is low, ice crystals only grow in soil pores^57,58^. This is consistent with our research results, the *K*_*s*_ of soil is significantly affected by changes in dry density and water content. Among them, compared with B7 and B9, C7 and C9, D7 and D9, E7 and E9, and F7 and F9, the *K*_*s*_ decreased by 42.25%, 53.52%, 57.36%, 49.80% and 41.15% respectively. Compared with B7 and B1, C7 and C1, D7 and D1, E7 and E1, and F7 and F1, the *K*_*s*_ increased by 17.0%, 22.57%, 7.83%, 9.26% and 24.07% respectively. In addition, it has been shown that external stress of up to 2 MPa can be generated during soil freezing^53^. Under the action of contact stress, the joints and frozen water in the soil will continue to grow and expand. As the circulation goes on, the growing ice crystals will produce pressure on the surface of the adjacent matrix and reduce the internal porosity of the soil^59^. Therefore, when the F–T cycle reaches 20 times, the *K*_*s*_ of the soil decreases instead. At the same time, the formation of ice-penetrating crystals increases the pore water pressure, compressing the soil, which in turn increases the pore water pressure. During the F–T cycle test, the soil was always inside the constraint ring, and with the increase of soil internal pressure, the constraint of the ring on the soil was strengthened. Considering that high water content promotes frost heave, this explains the crossover in the saturation conductivity curve.

### Pore size distribution of saline–alkali soil based on MIP after freeze–thaw cycles

Soil structure is an important factor affecting water conductivity^60–62^. The poor structure of saline–alkali soil is closely related to the low water conductivity and permeability of soil^63^, these are consistent with our experimental results. Soil microstructure is closely related to its *K*_*s*_ and is strongly affected by freeze–thaw cycles. In the test, A1, A2 and A3, as soil samples without freeze–thaw cycles, have porosity of only 6.19–11.51%. The low porosity resulted in the low saturated water conductivity of the soil sample, which was only 1.48 × 10^–5^ cm s^−1^. This makes it difficult for agricultural irrigation water to penetrate the soil, and the remaining water forms surface runoff, which accelerates soil nutrient loss. In addition, the dense soil structure and low porosity promoted soil capillarity in the floodplain of southwestern Shandong Province, and the salt remained on the surface with the evaporation of water after the rise of groundwater, which was extremely unfavorable to the development of agricultural production.

The MIP results showed that there were a lot of ultra-micropores in the soil samples in this area. However, more ultra-micropores in the soil were not conducive to the remediation of saline–alkali soil^49^. The F–T cycle can reduce the content of ultra-micropores and micropores in soil samples. After 10 F–T cycles, a new peak value appears at 10 μm, indicating that freeze–thaw cycles have a strong effect on soil macropores. When the number of freeze–thaw cycles reaches the threshold (*N* = 15), the pore water and ice crystals undergoing the freezing and thawing process applied pressure on the surrounding soil and ring knife. In addition, ice-permeable crystals formed during freezing and thawing, which can exacerbate the effects of pressure. Pressure caused particles to condense, which made the pore volume and diameter smaller at *N* = 20. These also caused the aperture distribution curves of D1 and F1 to coincide.

Water content and dry density have great influences on F–T cycle. The water transport process becomes more complex under freeze–thaw cycles, and the complexity increases with the increase of water content. In the soil sample, the water was almost completely frozen into ice during the freezing process, and the soil particles were displaced due to volume expansion, and the soil particles were not completely restored to the original position during thawing^64^, resulting in the deviation of the pore content and the increase of soil porosity. Therefore, we can determine that the increase of water content will shift the peak value of the curve to the right and increase the content of the intrusion volume, which is consistent with the results obtained from the saturated water conductivity test. In addition, the increase of dry density will strengthen the cementation between soil particles, and reduce the porosity in the soil. The growth of ice crystals and the formation of ice-penetrating crystals in the soil are further inhibited, the intrusion volume of pores is correspondingly reduced, and the saturated water conductivity of the soil is reduced.

### Effect of freeze–thaw cycles on salt discharge in saline–alkali soil

Under the action of freezing–thawing cycle, the saline–alkali soil was affected by the coupling of salt heaving and frost heaving, pore water and ice crystals, then pore channels were generated in the soil, which facilitated the flow of water and the frost heaving of ice. Therefore, in the early stage of freeze–thaw cycle, soil particles were broken, and the pore diameter and volume of soil became larger, correspondingly, *K*_*s*_ would increase. After the freezing–thawing cycle reached 10 times, the impact of freezing–thawing cycle intensified, leading to the crushing of large soil particles. The second peak of pore size distribution curve appeared. When *N* = 15, the pore volume reached the maximum, and then *K*_*s*_ reached the maximum. When *N* > 15, the pressure generated by ice crystals increase continuously, and the counterforce generated between the ice crystals and the soil matrix began to compress the soil interior. The internal pore channels of the soil were compressed, the pore volume and pore diameter correspondingly decreased, and *K*_*s*_ decreased. In addition, high water content could enhance the effect of ice crystals on soil, thereby increasing pore distribution. The higher dry density resulted in a denser interior of the soil sample, which reduced the flow of water through the channels.

In recent years, some scholars have tried to change the microstructure of saline–alkali soil with different ameliorants^65^. On this basis, we try to find a new ameliorating measure of saline–alkali soil. The former proposed that high soil porosity would improve its permeability^66^, and our research supports this view. The results show that the appropriate freeze–thaw cycle can greatly improve the pore distribution, soil porosity and saturated water conductivity of saline–alkali soil in the southwest of Shandong Province. This is also due to the weak internal cohesion of natural saline–alkali soil, and irreversible plastic deformation will occur after several times of frost heave, which will not be affected by the change of salt content. In a certain range, the increase of water content can enhance the effect of freeze–thaw cycle. Considering the natural water content of 14.07% and the optimal water content of 16% in the experimental area, this idea is feasible.

The extreme temperature in winter reaches − 16.5 °C in the Yellow Flood area of Southwest Shandong Province, which is a seasonal frozen soil area. Soil samples were taken from the topsoil layer (0–25 cm), which is included within the freezing depth of the soil in the area. Therefore, the F–T cycles can significantly improve the pore distribution and microstructure of crop surface soil. It is well known that after soil moisture evaporates, most of the salt and alkali remain in the surface of the soil^51^, which requires that we need to broaden the channels of water circulation in the soil and let the salt go with the water. Therefore, we can take the freeze–thaw cycle as the basis for the salt discharge in the saline–alkali soil project in this area. Based on the above research, in order to achieve the effect of improving the local soil microstructure and considering the influence of dry density on the pore distribution, we suggest that local farmers should properly plough the cultivated land in winter. Besides, burette can be used to increase the soil water content, in order to achieve the effect of promoting the freezing and thawing cycle.

## Conclusions

The existence of saline–alkali soil seriously endangers the agricultural production and development in the floodplain of southwest Shandong Province. In this experiment, MIP and SHC test were used to explore the relationship between soil microstructure characteristics and *K*_*s*_ under F–T cycles. The main reason of low permeability of saline–alkali soil is not the influence of grain size distribution and mineral composition. The low permeability of surface saline–alkali soil is closely related to its microstructure, and its pores are mainly composed of ultra-micropores and micropores. Under the action of F–T cycles, soil pores transition from micropores and ultra-micropores to small pores. When *N* = 10, mesopore particles begin to be broken, and such fragmentation is the most obvious when *N* = 15, which is consistent with the law presented by soil saturated hydraulic conductivity curve. High water content can improve the crushing effect of freeze–thaw cycle on soil particles, and then improve soil permeability, while the increase of dry density will play a certain inhibiting role. The results show that the permeability of saline–alkali soil is closely related to its microstructure, and the freeze–thaw cycle can improve the microstructure of saline–alkali soil to further improve its permeability, which provides a certain reference direction for the restoration of saline–alkali soil. Although the relationship between the microstructure of saline–alkali soil and the saturated hydraulic conductivity has been comprehensively analyzed by various methods in this experiment, there are still some limitations. In future studies, we need to consider the restoration effects of F–T cycles on other soil textures, and consider the coupling effects of F–T cycles on salt content, temperature and other factors in combination with soil microstructure.

## Data Availability

The data used to support the findings of this study are included within the article.
